# Iron Regulation of the Major Virulence Factors in the AIDS-Associated Pathogen Cryptococcus neoformans

**DOI:** 10.1371/journal.pbio.0040410

**Published:** 2006-11-21

**Authors:** Won Hee Jung, Anita Sham, Rick White, James W Kronstad

**Affiliations:** 1 The Michael Smith Laboratories, Department of Microbiology and Immunology, University of British Columbia, Vancouver, British Columbia, Canada; 2 Faculty of Land and Food Systems, University of British Columbia, Vancouver, British Columbia, Canada; 3 Department of Statistics, University of British Columbia, Vancouver, British Columbia, Canada; Duke University Medical Center, United States of America

## Abstract

Iron overload is known to exacerbate many infectious diseases, and conversely, iron withholding is an important defense strategy for mammalian hosts. Iron is a critical cue for Cryptococcus neoformans because the fungus senses iron to regulate elaboration of the polysaccharide capsule that is the major virulence factor during infection. Excess iron exacerbates experimental cryptococcosis and the prevalence of this disease in Sub-Saharan Africa has been associated with nutritional and genetic aspects of iron loading in the background of the HIV/AIDS epidemic. We demonstrate that the iron-responsive transcription factor Cir1 in *Cr. neoformans* controls the regulon of genes for iron acquisition such that *cir1* mutants are “blind” to changes in external iron levels. Cir1 also controls the known major virulence factors of the pathogen including the capsule, the formation of the anti-oxidant melanin in the cell wall, and the ability to grow at host body temperature. Thus, the fungus is remarkably tuned to perceive iron as part of the disease process, as confirmed by the avirulence of the *cir1* mutant; this characteristic of the pathogen may provide opportunities for antifungal treatment.

## Abstract 2

The authors identify an iron-responsive transcription factor in*Cr. neoformans* that controls the expression known major virulence factors.

## Introduction

The competition between host and pathogen for iron is a critical aspect of many infectious diseases including malaria, tuberculosis, and diarrheal diseases [1,2]. The availability of iron in host fluids is maintained at extremely low levels (10^−18^ M) by the iron-binding proteins transferrin (Tf) and lactoferrin (Lf). However, pathogenic microbes require 10^−6^ to 10^−7^ M iron for growth, and they must therefore steal iron from host proteins by binding ferrated Tf or Lf, elaborating siderophores, or degrading hemoglobin or other iron-containing proteins. Iron overload due to genetic predisposition, therapeutic intervention, or nutritional status is known to increase the risk of infection by many pathogens such as HIV, *Plasmodium falciparum, Mycobacterium tuberculosis,* and the fungal pathogen Cryptococcus neoformans [1,2].

*Cr. neoformans* is a basidiomycetous yeast that causes life-threatening meningoencephalitis in immunocompromised patients [3]. The major virulence factors of the two well-characterized varieties *neoformans* (capsule serotype D) and *grubii* (capsule serotype A) include the production of a polysaccharide capsule, the deposition of melanin in the cell wall, and the ability to grow at 37 °C. Acapsular mutants are avirulent, and the capsule has a variety of immunomodulatory affects, including inhibition of phagocytosis [4–8]. Capsule size is influenced by iron and CO_2_ levels, growth in serum, and host tissue location [9–12]. Melanin also influences phagocytosis and mediates resistance to oxidative stress [13]. The phenoloxidase (laccase) for melanin synthesis is required for virulence and is regulated by iron [14–16]. Tolerance to host temperature is also required for virulence, and the role of calcineurin in this phenotype has been well characterized [17–20].

Among fungi, iron transport and regulation are best understood in *Saccharomyces cerevisiae* [21]. Iron uptake is mediated by a high-affinity iron transport pathway in which ferric iron is reduced to ferrous iron by cell surface reductases (Fre1 and Fre2) and subsequently transported by the high-affinity iron permease/multicopper ferroxidase complex (Ftr1–Fet3). These and other components of the iron regulon are regulated by the transcriptional activators Aft1 and Aft2 [22,23]. Other fungi use transcriptional repressors to regulate the expression of iron-responsive genes [24]. Examples include Fep1 in *Schizosaccharomyces pombe,* Sfu1 in *Candida albicans,* and Urbs1 in Ustilago maydis. These proteins possess conserved cysteine-rich regions and two zinc finger motifs characteristic of GATA-type transcription factors in higher eukaryotes. Indeed, Fep1 and Urbs1 bind 5′-GATA-3′ sequences in the promoters of genes encoding high-affinity iron transporters, as well as siderophore production and transport functions [25–27]. *Cr*. *neoformans* is thought to acquire iron by both high- and low-affinity iron uptake systems mediated by cell surface reductases [28]. Nonenzymatic reduction of ferric iron by 3-hydroxylanthranilic acid and melanin has also been documented [29]. Genome-wide analysis of the response to low iron conditions using serial analysis of gene expression (SAGE) revealed that orthologs of many of the *Sa. cerevisiae* iron regulon genes (e.g., *FTR1, FET3,* and *FRE1*) are regulated by iron in *Cr. neoformans* [30,31]. However, regulators of iron-responsive genes have not yet been identified for *Cr. neoformans*.

In general, the regulatory mechanisms influencing iron transport and homeostasis are poorly understood in pathogenic fungi. Here we report the discovery of a gene that encodes a major transcriptional regulator of the response to iron in *Cr. neoformans*. This gene, *CIR1* (*Cryptococcus* iron regulator), shares structural and functional features with other fungal GATA-type transcription factors for iron regulation. For example, mutants lacking *CIR1* have elevated cell surface reductase activity as well as increased sensitivity to iron and phleomycin. Microarray analysis confirmed that *CIR1* influences the transcription of iron transport and homeostasis functions, as well as genes for calcium and cAMP signaling, and cell wall integrity. Parallel genetic analysis revealed that *CIR1* also controls the expression of all known virulence functions including capsule, melanin, and growth at host temperature. A similar link between iron and the expression of virulence factors (e.g., diphtheria toxin) occurs in bacterial pathogens [32,33], but the global association between iron and virulence in *Cr. neoformans* is remarkable. A *cir1* mutant was attenuated for virulence in a murine model of cryptococcosis, thus supporting the idea that iron regulation in *Cr. neoformans,* and perhaps in other fungi, is a promising target for antifungal therapy.

## Results

### Identification and Mutation of the *Cryptococcus* Iron Regulator, *CIR1*

**Figure 1:**
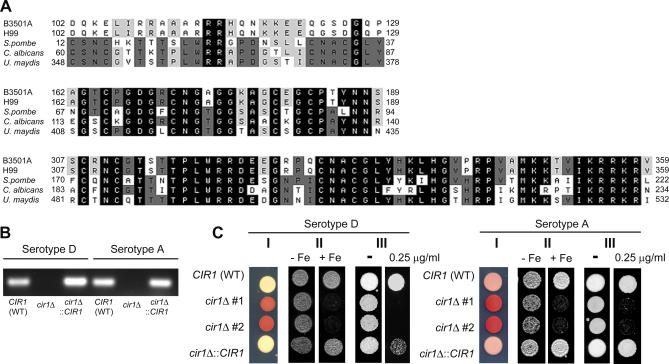
Conserved Regions of Cir1 and Iron-Related Phenotypes of *cir1* Mutants (A) Amino acid alignment of Cir1 with other fungal GATA-type iron regulators: *Sc. pombe* Fep1 (AAM29187), *Ca. albicans* Sfu1 (AAM77345) and U. maydis Urbs1 (AAB05617). Only the segments of the alignments containing the highly conserved N- or C-terminal zinc finger motifs (top and bottom alignments, respectively) and the cyteine-rich region are shown (middle alignment). Cir1 only has the C-terminal zinc finger motif. (B) RT-PCR results showing that *CIR1* transcripts are not produced from the *cir1* mutants, indicating complete disruption of the gene. WT. wild type. (C) Panel I, the *cir1* mutants display increased cell surface reductase activity as indicated by the red colony color in the presence of TTC; panel II, The *cir1* mutants are highly sensitive to elevated iron levels (+Fe, 0.75 mM ferrozine + 200 μM FeEDTA), but not to iron restriction (−Fe, 0.75 mM ferrozine); and panel III, the *cir1* mutants are more sensitive to phleomycin (0.25 μg/ml). Two independent mutants (#1 and #2) displayed the same phenotypes.

We initially used the sequences of the known fungal iron regulators Fep1, Urbs1, and Sfu1 to identify a candidate iron regulator, *CIR1,* in the genomes of serotype D and A strains of *Cr. neoformans* [31]. Sequences related to the *Sa. cerevisiae* Aft1 polypeptide were not found. A single copy of *CIR1* was identified in the serotype D and A strains, and these genes encoded predicted polypeptides of 963 (serotype D) and 952 (serotype A) amino acids (aa) with 93.4% aa identity. The Cir1 sequence was aligned with the fungal GATA-type iron regulators and found to share a zinc finger motif and a cysteine-rich domain (Figure 1A). However, unlike the other fungal iron regulators that have two zinc finger motifs, Cir1 contained only one zinc finger motif (the C-terminal motif), suggesting it may have different properties compared to other fungal iron regulators. We deleted the entire *CIR1* coding region in strains representing the D (strain B3501A) and A (strain H99) serotypes and found that the resulting *cir1* mutants were viable and had similar doubling times in YPD medium at 30 °C compared to wild-type cells (unpublished data). The *cir1* mutations in each strain were also complemented by integration of the wild-type gene of each serotype at the *CIR1* locus. The absence of *CIR1* transcript in the mutants was confirmed by RT-PCR (Figure 1B).

### *Cir1* Mutants Have Iron-Related Phenotypes

The *cir1* mutants were tested for iron-related phenotypes, including cell surface reductase activity, as well as sensitivity to iron and the iron-dependent inhibitor phleomycin. In *Sc. pombe,* loss of the transcriptional repressor Fep1 results in elevated cell surface reductase activity, which appears as a red colony color on media with triphenyltetrazolium chloride (TTC) [26]. As expected, colonies of the *cir1* mutants also showed an enhanced red color compared with wild-type strains, indicating higher cell surface reductase activity, presumably due to derepression of reductase gene transcription. The mutants complemented with *CIR1* had the wild-type phenotype, indicating that the increased reductase activity was due to deletion of *CIR1* (Figure 1C). Excess iron is potentially damaging because it can catalyze the formation of reactive oxygen species via the Haber-Weiss/Fenton reaction [34]. Additionally, the glycopeptide antibiotic phleomycin causes DNA damage in the presence of ferrous iron and oxygen due to the production of reactive species, and *sreA* and *fep1* mutants of Aspergillus nidulans and *Sc*. *pombe,* respectively, showed increased sensitivity to the drug [26,35–37]. In this regard, we found that the *cir1* mutants displayed sensitivity to excess iron and to phleomycin, indicating that *CIR1* is required for iron homeostasis (Figure 1C). As described below, the *cir1* mutants also displayed virulence-related phenotypes, including poor growth at 37 °C, loss of capsule formation, and altered melanin production. Taken together, these results linked Cir1 to iron-related phenotypes and virulence, thus warranting an examination of the influence of *cir1* mutation on transcription.

### Cir1 Plays a Major Role in the Transcriptional Regulation of Iron-Responsive Genes

For microarray experiments, wild-type and mutant strains were grown in low- or high-iron medium to analyze the transcriptional changes influenced by iron and by deletion of *CIR1*. A loop design was used with microarrays containing 70-mer oligonucleotides for 7,738 genes of the serotype D strain JEC21 (arrays are currently available only for this strain). We found that 733 genes were differentially expressed more than 2-fold in the wild-type strain (based on Q-value statistics). Among these, 483 genes were down-regulated, and 250 genes were up-regulated in low-iron versus high-iron medium, including genes related to iron transport and homeostasis, such as the high-affinity iron permease *(FTR1)* and the siderophore transporter *(SIT1)*. In general, there was good agreement with the genes found to be regulated by iron in our previous SAGE study [30]. In contrast to the situation with wild-type cells, there were no differentially expressed genes with statistical significance based on *q*-values in the *cir1* mutants in response to iron availability (*cir1*Δ; low vs. high iron; Table 1). A less stringent statistical evaluation based on *p*-values revealed a small number of genes that showed differential expression in the mutant in response to iron. In contrast, the comparisons between the wild-type strain and the *cir1* mutant revealed substantial differences in the transcriptomes in both low-iron and high-iron conditions. When the wild-type transcriptome was compared to that of the *cir1* mutant, 2,311 and 1,623 genes were differentially expressed in low-iron and high-iron media, respectively. Overall, these results indicated that Cir1 is a sensor of iron levels for *Cr. neoformans* and a key regulator of the corresponding transcriptional response.

**Table 1:**
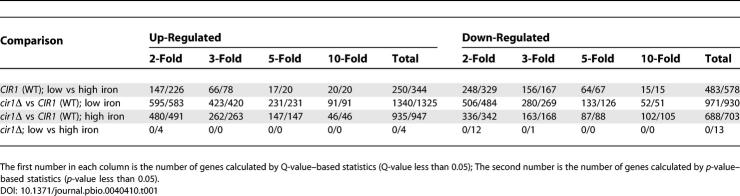
Number of Genes Differentially Regulated by Iron Availability and/or Cir1

We further analyzed the microarray data based on Gene Ontology (GO) categories for the biological processes of the differentially expressed genes, using the recently developed data-mining tool, ermineJ [38,39]. By using a gene score re-sampling analysis tool with all *q*-values from the experiments as input scores, we asked which GO categories were most enriched in the differentially expressed genes. The GO terms of iron ion transporter and siderophore transporter were the two most highly ranked groups, indicating that these genes were the most affected by both iron availability and disruption of *CIR1* (Table 2). Thus the ermineJ analysis supports the conclusion that *Cir1* functions in iron transport and homeostasis. Genes related to DNA replication, metabolism, and repair were also among the top-ranked groups; this is consistent with the sensitivity of the *cir1* mutants to excess intracellular iron, because transition metals such as iron are known to provoke the free radical–induced DNA damage [40]. Additionally, genes related to fatty acid metabolism and external encapsulated structure were affected by iron availability and/or disruption of *CIR1,* indicating (as described further below) that Cir1 has roles in membrane synthesis and cell wall integrity.

**Table 2:**
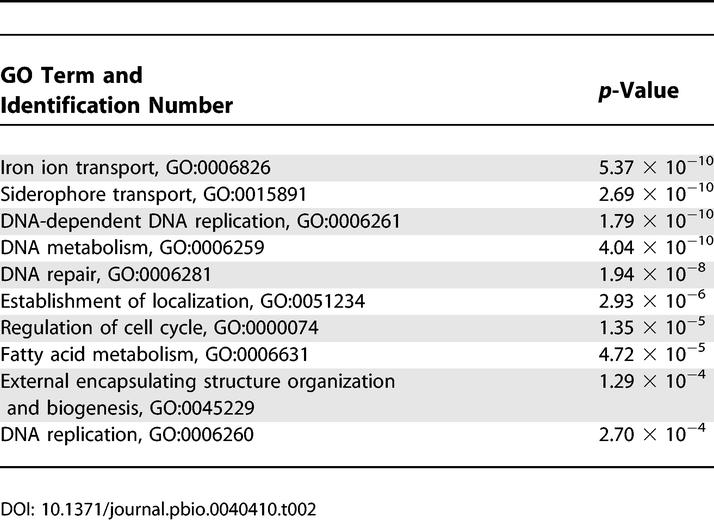
The Top Ten GO Terms Identified by Gene Score Re-sampling

### Cir1 Regulates Transcript Levels for Genes That Define the Iron Regulon

**Figure 2:**
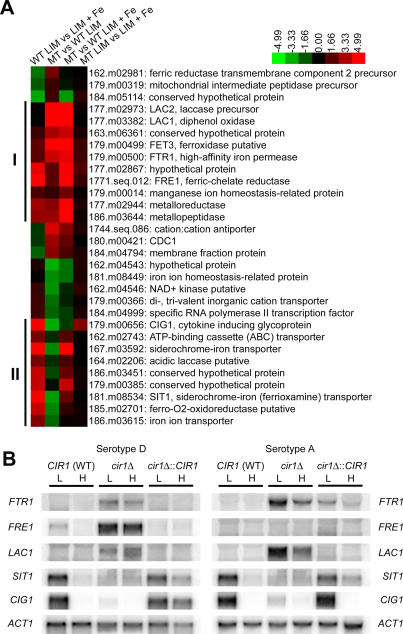
Cir1 Directly Regulates Genes Required for Iron Transport (A) Cluster analysis of genes required for iron transport show two patterns of differential expression in the *cir1* mutant (serotype D), resulting in two main clusters (I and II). Columns represent the log-transformed ratio of the array data from the wild-type strain in low- versus high-iron medium (WT LIM vs. LIM + Fe), the *cir1* mutant versus the wild-type strain in low-iron medium (MT vs. WT LIM), the *cir1* mutant versus the wild-type strain in high-iron medium (MT vs. WT LIM + Fe), and the *cir1* mutant in low- versus high-iron medium (MT LIM vs. LIM + Fe). (B) RNA blot analysis to confirm differential expression of genes required for iron transport in both the serotype D and the serotype A strains grown in a low-iron medium (L) or high-iron medium (H). WT, wild type.

Differentially regulated genes with GO terms related to iron transport and siderophore transport were extracted from the microarray data and clustered to identify candidate Cir1 targets and patterns of regulation. We found two main clusters of genes with higher transcript levels in response to iron limitation in wild-type cells, a pattern consistent with roles in iron transport (Figure 2A). Cluster I included genes related to reductive iron uptake systems such as *FTR1, FET3,* and *FRE1*. Transcript levels for genes in this cluster were also higher in the *cir1* mutants, suggesting negative and perhaps direct regulation by Cir1. Interestingly, the *LAC1* and *LAC2* genes encoding laccase for melanin production appeared in cluster I. This result is consistent with a potential role for laccase/melanin in reductive iron metabolism and revealed that Cir1 regulates one of the major virulence factors of *Cr. neoformans*. Phenotypic confirmation of this result is described below. A separate cluster (II) contained several genes for putative siderophore transporters, including *SIT1,* which was recently characterized as a siderophore transporter (K. Tangen, W. Jung, A. Sham, T. Lian, and J. Kronstad, unpublished data). Transcript levels for the genes in cluster II were decreased in the *cir1* mutant, especially in low-iron medium, thus raising the possibility that Cir1 may function both as a transcriptional repressor and an activator.

The microarray data were confirmed by RNA blot hybridization with selected genes from the serotype D strain B3501A, and we extended the analysis to include the same genes from the serotype A strain H99 for comparison. In general, the expression patterns matched the results from the microarray experiments (Figure 2B). For example, the *FTR1* transcript was clearly elevated in the *cir1* mutant relative to the wild-type or reconstituted strains for both serotypes (Figure 2B). The *FRE1* and *LAC1* genes showed a similar pattern of regulation, with higher transcript levels in the *cir1* mutant. These results support the placement of these genes in cluster I (Figure 2A) and suggest that Cir1 negatively regulates their expression. The hybridization results with *SIT1* and *CIG1* (encoding an iron-regulated cell wall protein; [30]) illustrate the pattern for cluster II genes in which loss of Cir1 results in lower transcript levels. We noted that a transcript signal was not found for the *FRE1* gene from strain H99, and this may indicate a divergence in regulation between the strains or serotypes. It is possible that other ferric reductase genes may be more important and more highly regulated in strain H99 than the one chosen for this analysis. Additionally, *FTR1* and *LAC1* in the *cir1* mutant of H99 (serotype A), but not in the mutant of B3501A (serotype D), clearly showed differential regulation by iron, implying that other regulators besides Cir1 may control their transcription and that the two serotypes may have different regulatory mechanisms. Overall, these results suggest that Cir1 negatively regulates expression of the reductive iron transport pathway and positively regulates iron-uptake systems mediated by siderophore transporters in *Cr. neoformans.*

### Cir1 Is Required for Elaboration of the Polysaccharide Capsule

**Figure 3:**
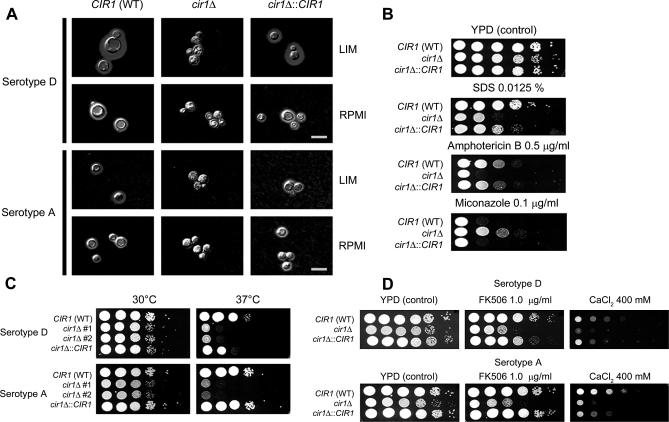
The *cir1* Mutants Are Defective in Expression of Major Virulence Factors (A) Strains were grown in low-iron medium (LIM) or RPMI medium at 37 °C under 5% CO_2_. Photographs were taken after 24-h incubation and after negative staining using India ink to visualize the capsule. Scale bars denote 10 μm. WT, wild type. (B) The *cir1* mutants of the serotype D strain displayed increased sensitivity to SDS and the antifungal drug amphotericin B. Decreased sensitivity was found to miconazole. (C) The *cir1* mutants of both serotype backgrounds displayed a growth defect at 37 °C; two independently generated mutants show the same phenotype. (D) Sensitivity of the *cir1* mutants to FK506 and CaCl_2_ was tested, and mutants in both serotype backgrounds displayed increased sensitivity compared to the wild-type strains.

The polysaccharide capsule is the major virulence factor of *Cr. neoformans* [11,12]. Given that iron limitation results in an enlarged capsule, we evaluated the effect of *CIR1* disruption on capsule formation by growing cells in low-iron medium. No capsule was visible on cells of the *cir1* mutants of both the serotype D and serotype A strains, whereas the wild-type and reconstituted strains displayed large capsules (unpublished data). Capsule formation was also evaluated by growing the cells in the presence of 5% CO_2_ at 37 °C to mimic the mammalian host environment. Elevated CO_2_ is known to induce capsule formation, but the *cir1* mutants still showed defective capsule formation under these conditions (Figure 3A). These results demonstrate that Cir1 is necessary for capsule formation, perhaps through the regulation of genes needed for sensing iron and CO_2_ levels, or for the synthesis, transport, or attachment of capsule components.

The loss of capsule production in the *cir1* mutants prompted us to examine the microarray data for insights into potential targets of Cir1 regulation that might account for this phenotype. We first compiled a list of genes related to signaling pathways and other components known to influence capsule and virulence (Table 3). We then examined the influence of iron and loss of Cir1 on the expression of these genes, with particular attention to targets like the *CAP* and *CAS* genes that are known to influence capsule formation [11,41]. Many of these genes were differentially expressed in the *cir1* mutants, but the changes were not significant. The *CAS32* gene was the exception, with 9.77- and 11.44-fold up-regulation in the *cir1* mutants in low-iron and high-iron media, respectively. This gene and other *CAS* family genes are homologs of the *CAP64* gene that is required for capsule formation; however, the *CAS* genes are involved in xylose branching and/or O acetylation of the capsule polysaccharide rather than capsule formation per se [41]. Therefore, it is not clear whether transcriptional changes in these capsule-related genes caused the capsule defect in the *cir1* mutants.

**Table 3:**
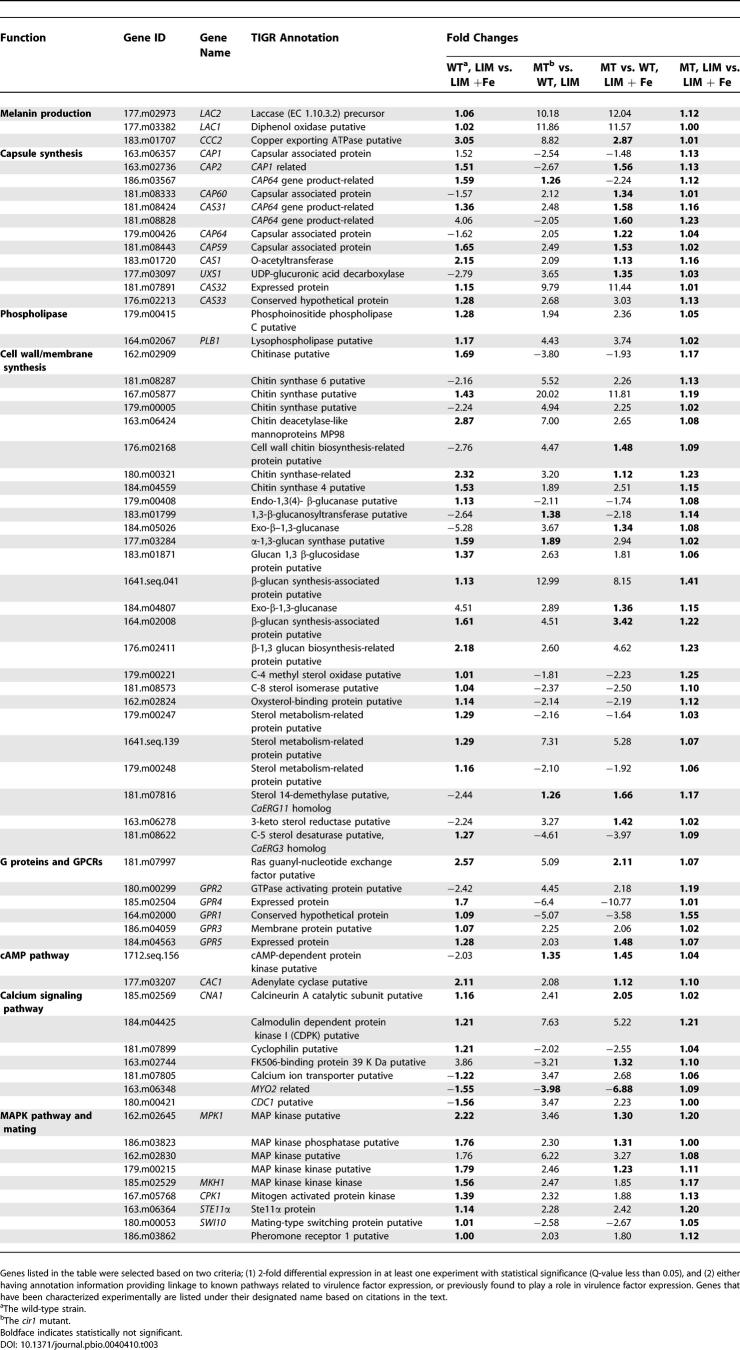
List of Genes Differentially Expressed and Related to Virulence Factor Expression

We also evaluated components of the cAMP pathway that positively regulates both capsule synthesis and melanin production in *Cr. neoformans* and found that transcripts for genes encoding adenylate cyclase or the catalytic subunit of PKA showed only subtle differences (Table 3; [42–44]). However, expression of the upstream G protein–coupled receptor (GPCR) Gpr4, which appears to activate Gpa1 in the cAMP pathway [45], was significantly reduced in the *cir1* mutants with 6.4- and 10.77-fold down-regulation in low-iron and high-iron conditions, respectively, compared to wild-type cells. Because *gpr4* mutants are defective in capsule synthesis, the loss of capsule in the *cir1* mutants could be due to down-regulation of this upstream receptor of the cAMP pathway. To test this hypothesis, the *cir1* mutants were grown in low-iron medium containing 10 mM cAMP. This resulted in only slight restoration of capsule formation in the *cir1* mutants of the serotype A strains (less than 20% of wild-type capsule size; unpublished data), but not in the *cir1* mutants of the serotype D strains. Therefore, we conclude that the deficiency in capsule synthesis of the *cir1* mutants is likely caused by alterations in other pathways in addition to the cAMP pathway.

Melanin production and glucose sensing were shown to be independent of Gpr4, indicating the potential involvement of other receptors [45]. Genes for other GPCRs were also differentially regulated in the *cir1* mutants, including *GPR1,* which was down-regulated in the *cir1* mutants in both low-iron and high-iron conditions, and *GPR2* and *GPR3,* which were up-regulated in the mutants in both conditions. No obvious phenotypes have been associated with these genes, and it is not clear that they influence the phenotypes of the *cir1* mutants [45]. Taken together, these data revealed that the membrane receptors of the cAMP pathway, but not the downstream effectors, were influenced by deletion of *CIR1*.

### Cir1 Influences Cell Wall Integrity and Membrane Functions

The cell wall is an important interface between *Cr. neoformans* and the host, and our earlier SAGE analysis of the response to iron limitation identified genes for wall components [30]. We therefore tested the *cir1* mutants for sensitivity to agents that challenge cell wall integrity and found reduced growth of the *cir1* mutant in the serotype D strain background on medium containing SDS (Figure 3B). We also examined the microarray data and found that the transcripts for genes encoding functions for the regulation, synthesis, and modification of chitin and glucan were generally increased in the *cir1* mutants (Table 3). Components of the Mpk1 (PKC1) MAPK pathway have been shown to regulate cell wall integrity in *Cr. neoformans* [46,47], and our microarray data revealed that the transcript of *MPK1* was 3.46-fold up-regulated in the *cir1* mutant compared to wild type in low-iron medium. We also found a gene for another MAP kinase (162.m02830) with high similarity to Mpk1 (55% identity and 70% similarity) on the same chromosome (Chromosome 9), and the transcript of this gene was significantly higher in the *cir1* mutants (6.22- and 3.21-fold in low-iron and high-iron conditions, respectively). These results support the idea that cell wall integrity is challenged by loss of Cir1 (Figure 3B) and that the Mpk1 MAP kinase pathway may be over-activated as a consequence. Components of other MAPK pathways in *Cr. neoformans* were also examined, and we found that disruption of *CIR1* caused increased transcript levels for two components of the mating pathway: *CPK1* and *STE11α* (an upstream MPKKK of Cpk1). Taken together, these results suggest that Cir1 may influence the cell wall through more than one mechanism, including the regulation of genes for biosynthetic enzymes and signaling components.

The microarray analysis implicated Cir1 in the regulation of genes for ergosterol synthesis, a result consistent with the increased sensitivity of the *cir1* mutant to the antifungal drug amphotericin B and the decreased sensitivity to miconazole that we observed (Figure 3B). Cir1 may play a role in remodeling sterol composition, perhaps through an influence on the iron-containing enzymes known to be required for sterol and unsaturated fatty acid biosynthesis. Furthermore, the transcript for *AFR1,* which encodes an ABC transporter protein responsible for azole resistance [48], was 3.2- and 4.64-fold up-regulated in the *cir1* mutants in low- and high-iron conditions, respectively (unpublished data). This result suggests that Cir1 may also influence anti-fungal resistance through regulation of membrane transporters. We should note, however, that the *cir1* mutant in strain H99 (serotype A) did not display altered sensitivity to SDS or the antifungal drugs tested, suggesting that there are strain or serotype differences in the influence of Cir1 (unpublished data). A number of phenotypic differences have been reported for serotype A and D strains, including differences in the phenotypes controlled by signaling pathways [44,49]. Two additional membrane-related genes that showed regulation by Cir1 encoded a putative phospholipase C and a lysophospholipase *(PLB1)*. The negative regulation of *PLB1* by Cir1 is interesting because this gene is required for full virulence in strain H99 [50]. The *PLB1* transcript was elevated 4.43-fold and 3.74-fold in the *cir1* mutant in low-iron and high-iron conditions, respectively (Table 3). It was previously reported that Plb1 activity is negatively regulated by another zinc finger protein Ste12α in a serotype D strain, and we hypothesize that there might be coordinate regulation with Cir1 [51,52].

### Cir1 Links Temperature- and Calcium-Sensitive Growth with Iron Homeostasis

We next examined the ability of the *cir1* mutants to grow at host temperature, because this is a critical virulence trait. The growth of the *cir1* mutants resembled the wild-type and reconstituted strains at 30 °C, but the mutants displayed a marked growth defect at 37 °C (Figure 3C). Cir1 may directly or indirectly regulate genes related to temperature stress, potentially in conjunction with calcium/calcineurin signaling in *Cr. neoformans* [17–19]. This idea is supported by our array data, which identified differentially expressed genes for the calcineurin catalytic subunit *(CNA1)* and for the following putative proteins: cyclophilin, FK506-binding protein, calmodulin dependent protein kinase (CDPK), and a calcium ion transporter (Table 3). Transcript levels of *MYO2* and *CDC1* were also affected by deletion of *CIR1*. These genes are interesting because of their relationship with calcium homeostasis in *Sa. cerevisiae* and because *MYO2* is a putative downstream target of the calmodulin pathway in *Cr. neoformans* [20,53]. We confirmed that calcium homeostasis was altered in the *cir1* mutants, by demonstrating increased sensitivity to exogenous CaCl_2_ and FK506 (Figure 3D). These results revealed important links between calcium and iron regulation.

### Cir1 Negatively Regulates Laccase Expression

**Figure 4:**
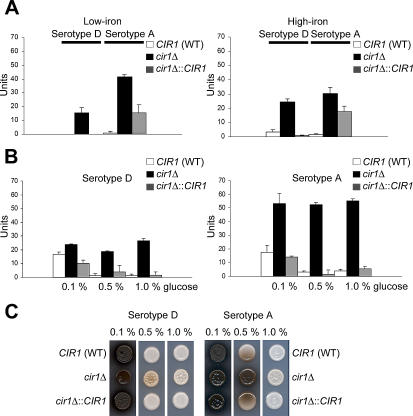
Cir1 Negatively Regulates Laccase Activity (A) Strains were grown in LIM and LIM + Fe under the same conditions used for the microarray experiments (0.5% glucose), and laccase activity was measured as described in Materials and Methods. WT, wild type. (B) Strains were grown in LIM containing different concentrations of glucose as indicated, and laccase activity was measured. All experiments were repeated three times, and averages are indicated with a bar (standard deviation). (C) Laccase activity of the wild-type, mutant, and reconstituted strains was compared by growing 1.0 × 10^5^ cells on DOPA plates with different concentrations of glucose as indicated. Photographs were taken after 3 d of incubation at 30 °C.

As noted earlier, the *LAC1* and *LAC2* genes were part of a cluster of genes encoding high-affinity and reductive iron-uptake functions, thus suggesting a possible role for laccase in related activities (Figure 2A). The marked up-regulation of *LAC1* in the *cir1* mutant in both low- and high-iron media prompted an analysis of laccase activity under the same conditions. As shown in Figure 4A, laccase activity was indeed increased in the *cir1* mutants compared to wild-type cells, in agreement with the microarray experiments. We also noticed that the activity of laccase in the serotype A strain (H99) was generally higher than in the serotype D strain (B3501A), further reinforcing the differences between the strains and suggesting a possible contribution of laccase activity to the relatively higher virulence of the H99 strain. We investigated whether the elevated laccase activity in the *cir1* mutants was affected by the glucose concentration in the medium, because glucose is known to repress expression [14]. Laccase activity appeared to be constitutive in the *cir1* mutants with regard to glucose concentration (0.1%, 0.5%, and 1.0%; Figure 4B). As expected, the wild-type and reconstituted strains displayed derepression of laccase activity only in media containing 0.1% glucose. Similarly, constitutive de-repression of laccase in the *cir1* mutants was observed on DOPA medium with different glucose concentrations (Figure 4C). Finally, we noted a higher transcript level for the gene encoding the copper transporter Ccc2 in the *cir1* mutant, indicating negative regulation by Cir1 (Table 3). Ccc2 is required for melanization in *Cr. neoformans,* possibly through an influence on intracellular copper concentration [54]; this role for Ccc2 may also contribute to the increased laccase activity of the *cir1* mutant. We conclude that Cir1 is a negative regulator of laccase expression and that Cir1 is required for the influence of glucose on laccase expression.

### Loss of Cir1 Abolishes Virulence

**Figure 5:**
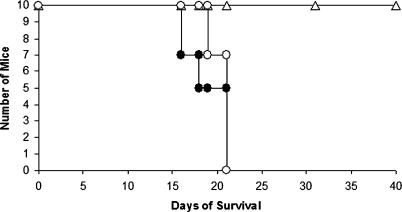
The *cir1* Mutants Are Attenuated for Virulence Ten female A/Jcr mice were infected intranasally with the wild-type, serotype A strain H99 (filled circle [•]), a serotype A *cir1* mutant strain (open triangle [Δ]) or a reconstituted strain (open circle [○]). The survival of the mice is shown versus time in days. The assay was repeated twice with two independently generated mutants, and a representative result is shown.

The accumulation of virulence-related phenotypes for *cir1* mutants strongly suggested a defect in virulence, and we tested this prediction in the mouse inhalation model of cryptococcosis. We tested the *cir1* mutant in the serotype A strain H99 because this background displays the highest virulence, and we found that the mutant was avirulent. In contrast, mice infected with the parental or reconstituted strains succumbed to infection by approximately 20 d (Figure 5). These results strongly support our hypothesis that Cir1 is a key regulator of virulence gene expression, and demonstrate the importance of iron regulation for cryptococcosis.

## Discussion

**Figure 6:**
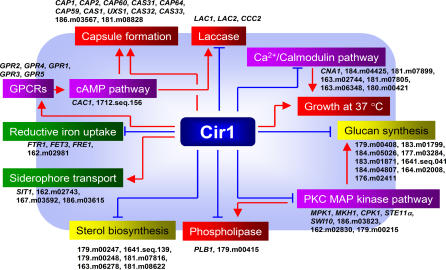
Cir1 Is a Central Regulator of the Iron Regulon, Virulence Factor Expression, and Virulence-Associated Signaling Pathways A schematic of the functions controlled by Cir1 is shown to indicate positive and negative regulation, and interconnections between signalling pathways and downstream target functions. Red arrows indicate positive regulation by Cir1, and blue blunt arrows indicate negative regulation by Cir1. The genes beneath each functional box are downstream targets of Cir1 as experimentally determined by the present study and as listed in Table 3. Gene names are listed for those that have already been characterized; TIGR gene identifiers are used for the other genes.

The mechanisms by which fungal pathogens sense the mammalian host environment and regulate virulence factor expression are poorly understood. To address this issue, we identified Cir1 as a candidate iron-responsive transcription factor in *Cr. neoformans,* and characterized phenotypic and transcriptional changes resulting from deletion of the gene. Remarkably, we found that Cir1 regulates the majority of iron-responsive genes and influences all of the major known virulence factors (summarized in Figure 6). Specifically, the transcript levels for 733 genes changed in wild-type cells in response to iron concentration. Of these, 250 were up-regulated in low-iron versus high-iron medium, and deletion of *CIR1* largely eliminated the response of this group of genes. Key iron-related genes that were negatively regulated by Cir1 included components of the reductive uptake system (i.e., *FRE1, FTR1,* and *FET3*). Orthologs of some of these genes are also negatively regulated by Sfu1 in *Ca. albicans* and by Fep1 in *Sc. pombe* [26,27,55]. Candidate siderophore transporter genes are also negatively regulated by Fep1 and Sfu1 [27,55] but, in contrast, these functions appeared to be positively regulated by Cir1 suggesting that the protein may function both as a repressor and an activator to regulate different iron transport systems. Lan et al. also found that Sfu1 has both positive and negative regulatory functions, and interestingly, some of the positively regulated functions included cell-surface components [55]. Structural differences between Cir1 (e.g., single zinc finger motif) and the other fungal iron regulators (two zinc finger motifs) may account for variation in regulatory capabilities.

The positive influence of Cir1 may result from downstream regulators that transcriptionally or post-transcriptionally control the genes for siderophore transporters and other functions. In *Sa. cerevisiae,* the iron regulators Aft1 and Aft2 control expression of Cth2, which in turn enhances mRNA decay for transcripts of genes of the TCA cycle as well as sterol and heme biosynthesis [56]. This study and work in bacteria and mammalian cells reveal that post-transcriptional regulation is an important component of the cellular response to iron deprivation [57,58]. Although additional levels of iron regulation have not yet been identified in *Cr. neoformans,* we hypothesize that the positive and negative roles of Cir1 might allow modulation of iron acquisition systems in response to different iron sources available in the host or the environment. It is interesting to note that Cir1 shares regulatory features with the iron-responsive transcription factors such as Fur (Ferric uptake regulator) in bacterial pathogens [32,33]. One feature in common is that Fur can exert both positive and negative regulation, as demonstrated in *Escherichia coli, Helicobacter pylori, Neisseria meningitides,* and Vibrio cholerae [59–63]. Moreover, bacterial Fur proteins not only influence iron uptake/homeostasis, but also several other cellular processes including pyrimidine metabolism, methionine biosynthesis [64], nonfermentable carbon source utilization [65], acidic tolerance [66], oxidative stress [60], chemotaxis [67], and virulence factor (e.g., shiga toxin and diphtheria toxin) expression [68]. The diversity of these processes is reminiscent of the features of Cir1 regulation revealed by our expression studies (Figure 6).

Our analysis of Cir1 revealed a link between iron regulation and melanin production, an important virulence trait in *Cr. neoformans* [13,69–71]. The neurotropism of *Cr. neoformans* may result in part from the ability of the fungus to produce protective melanin from catecholamines in the brain [14]. A recent study demonstrated that laccase is expressed early in murine infection (until 24 h) and decreases thereafter as the fungal burden rises [72]. The same study showed that laccase is released from the cell wall in vivo, and suggested a role for laccase as an antioxidant or an iron scavenger. Lui et al. also proposed iron-related functions for laccase, based on the similarities of the copper-containing regions of laccase and Fet3 [73]. The enzyme does possess strong ferrous iron oxidase activity in the absence of substrates, and this activity may protect cells from hydroxyl radicals generated from host macrophages and neutrophils, and potentially contribute to iron transport [73]. Our observations support a role for laccase in iron metabolism, because *LAC1* and *LAC2* were identified as targets of Cir1 with expression patterns similar to genes of the reductive iron transport pathway (e.g., *FTR1* and *FET3*). We speculate that laccase oxidation of ferrous iron may be important during infection in addition to the enzyme's role in melanin production. The fact that laccase is expressed only during very early stages of infection would be consistent with a role in iron acquisition and in protection of fungal cells from hydroxyl radical attack from host cells during the initial adaptation to the host environment. Additionally, we found putative Cir1 binding sites (GATA motif) in the promoter region of *LAC1* (unpublished data), and interestingly, regions containing these motifs were previously proposed to bind to negative regulatory proteins [74]. Up-regulation of the copper transporter Ccc2 in the *cir1* mutant may increase intracellular copper concentration and partly contribute to higher laccase activity, because copper is known to induce transcription of *LAC1* [54,75].

The cell wall plays a key role in the virulence of *Cr. neoformans* as the site of melanin deposition and capsule attachment [11,12,76]. We found that Cir1 and iron may influence cell wall remodeling and capsule formation by at least two mechanisms. First, the transcription of genes related to cell wall functions, including chitin synthases and α-1,3-glucan synthase, was altered in the *cir1* mutants, and the mutants have defects in cell wall integrity. Additionally, Cir1 influences expression of the MAP kinase of the cell wall integrity pathway *(MPK1)*. Capsule polysaccharide attachment to the cell surface requires α-1,3-glucan [76] and deletion of *CIR1* may alter glucan composition to influence attachment. A second mechanism may involve Cir1 regulation of the cAMP pathway that controls capsule and melanin formation [43]. We found that the transcript for Gpr4 is positively controlled by Cir1, and Xue et al. have shown that the capsule size of the *gpr4* mutants is reduced by 30% compared to the wild type [45]. In this study, exogenously added cAMP partially restored capsule on cells of the *cir1* mutants in the serotype A, but not the serotype D background. Together, these results suggest that Cir1 interacts with the cAMP pathway, but that the protein regulates other capsule-related functions. That is, the mechanisms for biosynthesis and assembly of the capsule are undoubtedly complex, and the capsule defect in *cir1* mutants likely is due to additional mechanisms. For example, changes in the redox status on the cell surface of the mutants could contribute to poor capsule assembly or attachment. Surface changes would be consistent with the higher levels of cell surface ferric reductase and laccase activity displayed by the mutants.

We found that loss of *CIR1* influences the transcript levels for components involved in calcium signaling (e.g., calcineurin), and an extensive body of elegant work connects these functions to virulence and the stress response in *Cr. neoformans* [77,78]. Additionally, Kraus et al. showed that inactivation of calcineurin causes an Mpk1-dependent increase in transcript level for the *FKS1* gene encoding a component of β-1,3-glucan synthase [46]. Our phenotypic analysis revealed that *cir1* mutants showed increased sensitivity to CaCl_2_ and FK506, as well as a growth defect at 37 °C, implying that Cir1 and calcium signaling components influence common targets. Connections between iron and calcium homeostasis could occur at a number of levels. For example, the up-regulation of a calcium transporter or a putative Ca^2+^/calmodulin-dependent protein kinase (type II) in the *cir1* mutant (Table 3) could influence sensitivity to calcium. The kinase is one of the downstream regulatory proteins activated by Ca^2+^/calmodulin along with calcineurin. It is also possible that elevated intracellular iron in the *cir1* mutants might directly influence the activity of calcineurin because this protein contains a Fe-Zn dinuclear metal center at its active site that is required for full activity [79].

Defects in host iron homeostasis exacerbate many bacterial, fungal, and parasitic infections [1,2,80]. For fungi, iron overload increases the mortality of mice infected with *Ca. albicans,* and elevated iron was found in patients with vulvovaginal candidosis [81,82]. Similarly, iron overload and chelation therapy with deferoxamine both enhance zygomycoses, thus illustrating the importance of a balance in iron availability [83]. Iron overload and other host factors such as smoking also exacerbate cryptococcal meningoencephalitis, perhaps by stimulating fungal growth and perturbing macrophage-mediated anticryptococcal defenses [84–86]. Our analysis of Cir1 reveals the underlying importance of iron sensing in the expression of virulence factors leading to cryptococcosis. These finding could have therapeutic value because the prevalence of cryptococcosis in sub-Saharan Africa may be associated with nutritional and genetic aspects of iron overloading in the background of the HIV/AIDS epidemic [87,88]. An understanding of the regulation of *Cr. neoformans* virulence could have considerable impact because cryptococcosis is responsible for 13%–44% of all deaths of HIV-infected patients in sub-Saharan Africa [89].

## Materials and Methods

### Strains and growth conditions.

Strains (Table S1) were grown in yeast extract, bacto-peptone, and 2.0% glucose (YPD; Becton, Dickinson and Company, Franklin Lakes, New Jersey, United States) medium or yeast nitrogen base (YNB, Becton, Dickinson and Company) with 2.0% glucose. Low-iron medium (LIM) was prepared as described [9]. Iron-replete medium (LIM + Fe) contained 100 μM of ethylenediaminetetraacetic acid ferric-sodium salt (FeEDTA; Sigma, St. Louis, Missouri, United States).

### Isolation of *CIR1* and mutant construction.

The *CIR1* genes in strains B3501A and H99 were identified by searching for homologs of *Sc. pombe* Fep1 in the serotype D genome database (TIGR Cryptococcus neoformans Genome Project [http://www.tigr.org/tdb/e2k1/cna1/]) or the serotype A genome database (Broad Institute Cryptococcus neoformans Database [http://www.broad.mit.edu/annotation/fungi/cryptococcus_neoformans/index.html]), respectively. The cDNAs of *CIR1* from B3501A and H99 were amplified by RT-PCR, cloned into pCR2.1 (Invitrogen, Carlsbad, California, United States), and sequenced.

To construct *cir1* mutants, the entire locus of *CIR1* in both B3501A (3,606 base pairs [bp]) and H99 (3,576 bp) was replaced by a disruption cassette containing the nourseothricin acetyltransferase gene *(NAT)* and 5′ and 3′ flanking sequences of *CIR1* were included for homologous recombination. The primers J2FEP-5F and J2FEP-5R, and J2FEP-3F and J2FEP3R were used to amplify the 5′ upstream (−911 to −11) and the 3′ downstream regions (+3,608 to +4,655) of B3501A *CIR1,* respectively (Table S1). The primers H9FEP-5F and H9FEP-5R (−1,024 to −33), and H9FEP-3F and H9FEP-3R (+3,805 to +4,806) were used to amplify the 5′ upstream and the 3′ downstream region of H99 *CIR1,* respectively (Table S1). Amplified 5′ and 3′ flanking regions from B3501A *CIR1* and H99 *CIR1* were digested with XhoI and ApaI, and, SpeI and SacI, respectively, and ligated to pCH233 containing the *NAT* gene to generate plasmids pWH008 and pWH016. Disruption cassettes from the plasmids were amplified by PCR using primers J2FEP-5F and J2-FEP3R, and H9FEP-5F and H9FEP-3R, and biolistically transformed into wild-type strains as described previously [90]. Positive transformants were identified by PCR and confirmed by Southern blot analysis (Figure S1). At least two independent mutants in each strain were used throughout the study.

To construct reconstituted strains, primers J2CIR-F-Xb and J2CIR-R-Nt, and H9CIR-F-Xb and H9CIR-R-Nt were used to amplify the wild-type *CIR1* genes from B3501A and H99, respectively (Table S1). These fragments were digested with XbaI and NotI and cloned to pCH233 to construct pWH020 and pWH021. The SacI- and SpeI-digested fragments from pWH008 and pWH016 were then cloned into pJAF to construct pWH023 and pWH024 containing the neomycin-resistant marker *(NEO)* and the 3′ downstream regions of *CIR1* of B3501A and H99, respectively. The *NEO*-*CIR1* 3′ region fusion fragments were released by digestion with NotI and KpnI, and were cloned into pWH020 and pWH021 digested with the same enzymes, respectively. The resulting plasmids pWH031 and pWH034 were digested with XbaI and transformed into the *cir1* mutants of B3501A and H99, respectively. Positive transformants containing the wild-type *CIR1* gene at its authentic locus were identified by PCR.

### Phenotypic analysis.

The overlay assay with TTC (Sigma) to evaluate cell surface reductase activity was performed as described previously [91,92]. To assess strain sensitivity to iron-restricted or to iron-overload conditions, 1.0 × 10^4^ cells were spotted onto YPD medium (containing 0.75 mM ferrozine) with or without 200 μM FeEDTA and were grown at 30 °C for 2 d. Phleomycin sensitivity was performed by spotting 1.0 × 10^4^ cells onto YPD medium containing 0.25-μg/ml phleomycin. For other plate assays, 10-fold serial dilutions of cells were spotted onto YPD plates containing chemicals as indicated, with incubation at 30 °C for 2 to 3 d. To assess capsule formation, cells were grown in YNB medium at 30 °C overnight, washed twice with low-iron water, and diluted to 5.0 × 10^6^ cells/ml in LIM or RPMI medium (Invitrogen). Cells were then grown for 24 h at 30 °C or 37 °C under 5% CO_2_.

### Laccase assay.

Cells were grown in YNB overnight at 30 °C, pelleted and then washed three times with LIM, diluted to 1.0 × 10^7^ cells/ml in 50 ml of LIM with 0.1%, 0.5%, or 1.0% glucose, and incubated for 12 h at 30 °C. After incubation, 1.0 × 10^7^ cells were withdrawn from each culture and then washed three times with LIM. Cells were then incubated in 10 mM L-DOPA (Sigma) for 30 min (B3501A) or 10 min (H99) at 30 °C. A shorter incubation time was applied for H99 because the significantly stronger melanin formation of the serotype A *cir1* mutants causes precipitation of melanin in the reaction tubes and interferes proper OD readings. Cells were pelleted at the end of the incubation and the absorbance (A_475_) of the supernatant was measured. The enzyme activity was calculated as A_475_ of 0.001 = 1 unit of laccase. DOPA medium was used for plate melanin assays as described [93,94].

### RNA hybridization and microarray experiments.

The *cir1* mutant B3CIR1#572 and the parent strain B3501A were used for microarray analysis. Three biological replicates for each strain were grown in 50 ml of YNB overnight at 30 °C, followed by growth in LIM at the same temperature for an additional 12 h. The latter step was added in order to eliminate any iron carryover from the rich medium. Cultures were harvested and then washed twice with LIM. Cell numbers were determined, and cells were transferred to 50-ml LIM or LIM + Fe (final density of 1.0 × 10^7^ cells/ml). Cells were grown at 30 °C for another 12 h, harvested, and lyophilized for RNA extractions. Cell densities at the time of harvest were 5.0 × 10^7^ cells/ml (wild type in LIM), 1.0 × 10^8^ cells/ml (wild type in LIM + Fe), 1.0 × 10^7^ cells/ml (*cir1*Δ in LIM), and 1.5 × 10^7^ cells/ml (*cir1*Δ in LIM + Fe). Trizol (Invitrogen) was used for total RNA extractions following the manufacturer's recommendations. RNA was analyzed with the Agilent 2100 Bioanalyzer (Agilent Technologies, Santa Clara, California, United States) and cDNA was synthesized from 5 μg of total RNA by SuperScriptII Reverse Transcript Enzyme (Invitrogen). The 3DNA Array 350 kit (Genisphere, Hatfield, Pennsylvania, United States) was used to label cDNA with Cy3 or Cy5 for hybridization to 70-mer microarrays (http://genome.wustl.edu/activity/ma/cneoformans/).

RNA blot analysis was performed as described by Sambrook et al. with 5 μg of total RNA from each strain [95]. Hybridization probes were designed for the genes from each serotype, and DNA fragments were amplified separately by PCR with the primers listed in Table S2. A Strip-EZ DNA kit (Ambion, Austin, Texas, United States) was used for probe labeling, and the membrane was exposed to a Phosphor Screen (Amersham Bioscience, Piscataway, New Jersey, United States) for 12 h and scanned using a Storm 860 Gel and Blot imaging system (Amersham).

### Microarray hybridization, statistical analysis, and data mining.

The following loop design, which consists of four nodes and paths including dye-swap, was adopted for this study: wild type (LIM) ↔ wild type (LIM + Fe), wild type (LIM) ↔ *cir1*Δ (LIM), wild type (LIM + Fe) ↔ *cir1*Δ (LIM + Fe), and *cir1*Δ (LIM) ↔ *cir1*Δ (LIM + Fe). A total of 24 arrays were used for the experiment. After hybridization, arrays were scanned immediately using the PerkinElmer ScanArray Express (PerkinElmer, Wellesley, California, United States). Each channel was background corrected by subtracting the lowest 10% of foreground signal intensity. The two channels of each array were normalized to each other by Huber's variance stabilization algorithm, *vsn* [96]. A linear mixed effects model was applied to the normalized data in each channel. A fixed effect was included for each array, for the dye by gene interaction, and for each combination of iron availability and/or deletion of *CIR1*. Random effects were included for within-array variability (each gene appeared twice on each array), technical variability (each replicate culture was hybridized four times), and biological variability (12 replicate cultures were employed). The changes in wild type (LIM) versus wild type (LIM + Fe), wild type (LIM) versus *cir1*Δ (LIM), wild type (LIM + Fe) versus *cir1*Δ (LIM + Fe), and *cir1*Δ (LIM) versus *cir1*Δ (LIM + Fe) were estimated with standard errors and *p*-values based on Student *t* statistics; *q*-values were computed to adjust for the false discovery rate.

The microarray data mining tool ermineJ was used to analyze the microarray dataset based on GO terms [39]. The *q-*values from the microarray data were used as input scores and gene score resampling analysis (GSA) was applied. For clustering, genes with GO terms (cellular process) related to iron transport and homeostasis, and that were more than 2-fold differentially expressed in at least one experiment (with a statistically significant *q*-value less than 0.05), were extracted from the microarray data. The signal ratio of these genes was then log-transformed, clustered by Cluster 3.0 (average linkage) [97], and visualized by Java Tree View 1.0.12 [98].

### Virulence assay.

Thirty female A/Jcr mice (4 to 6 wk old) were obtained from Jackson Laboratories (Bar Harbor, Maine, United States). The *Cr. neoformans* cells for inoculation were grown in YPD medium overnight at 30 °C, washed twice with PBS, counted with a haemocytometer, and resuspended at 1.0 × 10^6^ cells/ml in PBS. Mice were weighed and then anesthetized with ketamine and xylazine in saline. Mice were secured on a thread by their superior incisors, 50 μl of the cell suspension (5.0 × 10^4^ cells) was intranasally instilled, and the mice were left on the thread for 10 min. The status of the mice was monitored twice per day post-inoculation. The protocol for the virulence assays (protocol A99–0252) conformed to regulatory standards and was approved by the University of British Columbia (UBC) Committee on Animal Care.

## Supporting Information

Figure S1 Disruption of Wild Type *CIR1* Was Confirmed by Southern Blot Analysis Restriction maps of genomic regions containing a wild-type or a disrupted *CIR1* allele are shown. Genomic DNAs of serotype D and serotype A strains were digested with BamHI/HindIII and XhoI/HindIII, respectively, and hybridized with the probes indicated.
(A) Lane 1 represents serotype D wild-type strain B3501A. Lanes 2 and 3 represent the *cir1* mutant strains B3CIR572 and B3CIR672, respectively.
(B) Lane 1 represents serotype A wild-type strain H99. Lanes 2 and 3 represent the *cir1* mutant strains H9CIR4 and H9CIR24, respectively.
(1.2 MB TIF)

Table S1 Strains and Primers Used in This Study (34 KB DOC)

Table S2 Primers Used for RNA Blot Analysis (27 KB DOC)

### Accession Numbers

Cloned *CIR1* cDNAs of *Cr. neoformans* serotype D (B3501A) and serotype A (H99) were sequenced, and the results were submitted to GenBank (http://www.ncbi.nlm.nih.gov/Genbank) under accession numbers DQ631833 and DQ631834, respectively. The microarray data was submitted to Gene Expression Omnibus (GEO; http://www.ncbi.nlm.nih.gov/geo/) under accession number GSE5341. TIGR gene identifiers were used throughout the text based on the format of the microarray annotation file. The corresponding GenBank identifiers can be found at the TIGR database: http://www.tigr.org/tdb/e2k1/cna1/.

## Abbreviations

GO: Gene Ontology
Lf: lactoferrin
SAGE: serial analysis of gene expression
Tf: transferrin
