# Correction: Iron Regulation of the Major Virulence Factors in the AIDS-Associated Pathogen *Cryptococcus neoformans*

**DOI:** 10.1371/journal.pbio.1002410

**Published:** 2016-03-08

**Authors:** Won Hee Jung, Anita Sham, Rick White, James W Kronstad

There is an error in Fig 2B of the published article: the blots for SIT1 Serotype A and Serotype D are identical. Please view the correct Fig 2 here.

**Fig 2:**
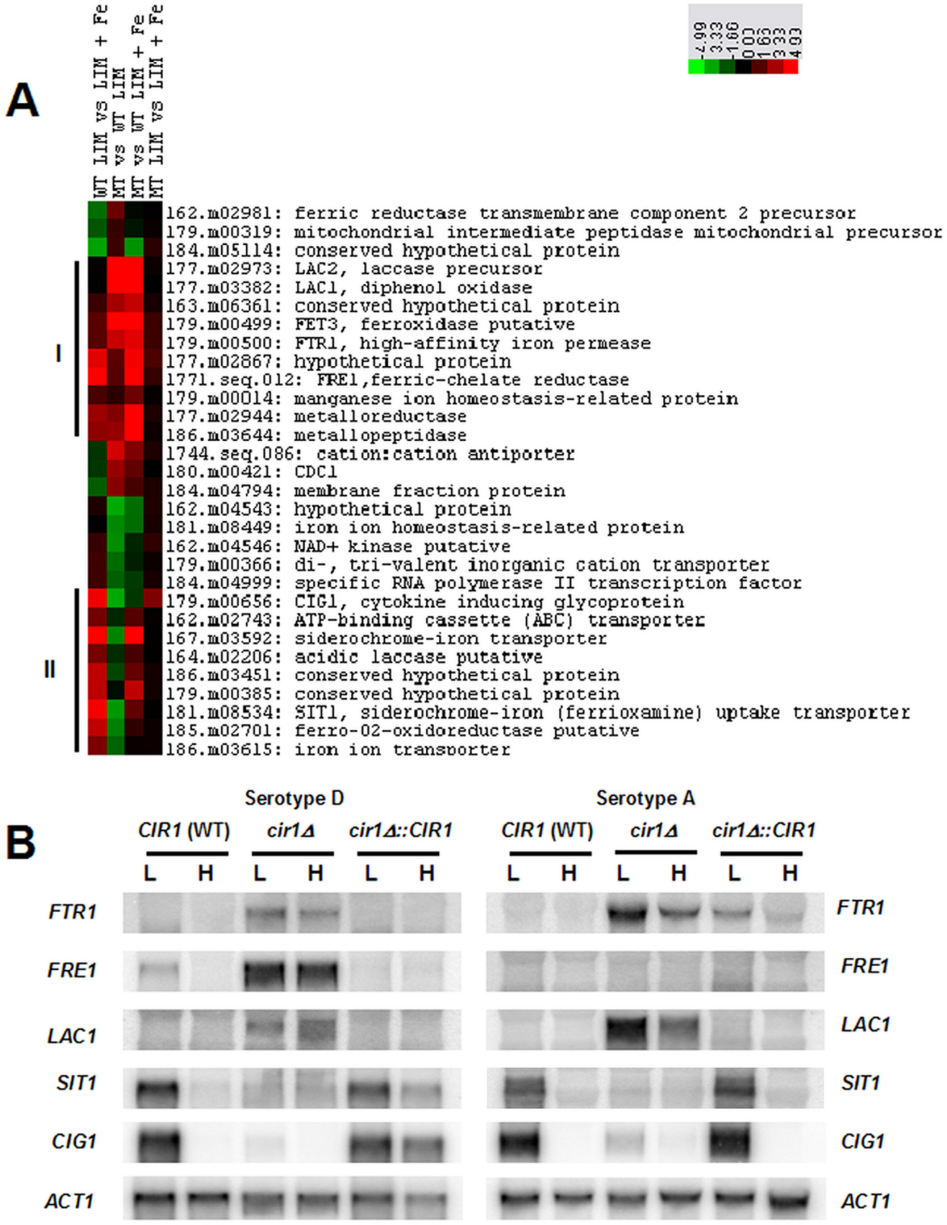
Cir1 Directly Regulates Genes Required for Iron Transport. (A) Cluster analysis of genes required for iron transport show two patterns of differential expression in the *cir1* mutant (serotype D), resulting in two main clusters (I and II). Columns represent the log-transformed ratio of the array data from the wild-type strain in low- versus high-iron medium (WT LIM vs. LIM + Fe), the *cir1* mutant versus the wild-type strain in low-iron medium (MT vs. WT LIM), the *cir1* mutant versus the wild-type strain in high-iron medium (MT vs. WT LIM + Fe), and the *cir1* mutant in low- versus high-iron medium (MT LIM vs. LIM + Fe).
