# Supplementary material for: Iron Regulation of the Major Virulence Factors in the AIDS-Associated Pathogen Cryptococcus neoformans
Source: PLoS Biol. 2006 Nov 21;4(12):e410. doi: 10.1371/journal.pbio.0040410 (PMC1637126; doi:10.1371/journal.pbio.0040410)
Supplement: Supplementary file 1 [file pbio.0040410.st001.doc]

#### Table S1. Strains and primers used in this study

| Strains | |  |  | |
| --- | --- | --- | --- | --- |
| Serotype | Name | Genotype | Reference or source | |
| D | B3501A | MATα | [1] | |
|  | B3CIR1572 | *MATα cir1::NAT* | This study | |
|  | B3CIR1672 | *MATα cir1::NAT* | This study | |
|  | B3CIR-R39 | *MATα cir1::NAT*, *CIR1-NEO* | This study | |
| A | H99 | MATα | [2] | |
|  | H9CIR4 | *MATα cir1::NAT* | This study | |
|  | H9CIR24 | *MATα cir1::NAT* | This study | |
|  | H9CIR-R13 | *MATα cir1::NAT*, *CIR1-NEO* | This study | |
|  |  |  |  | |
| Primers | |  |  | |
| Name | Sequence | | | |
| J2FEP-5F | GTAACGGGCCCTTCTTTCTCCATCACCTCCCCT | | | *Apa*I |
| J2FEP-5R | GTGGCCTCGAGAGCGAATCGTGCCATGGTAT | | | *Xho*I |
| J2FEP-3F | GAGTACTAGTTGGCACAGGCGACTAGTGATTA | | | *Spe*I |
| J2FEP-3R | GGTTGGGAGCTCCTTCACCTGGAACATGCGTC | | | *Sac*I |
| H9FEP-5F | GTGTGGGCCCTGGCGTACTGTATCAATGTCAA | | | *Apa*I |
| H9FEP-5R | GGTTCCCTCGAGAACCATGCGTATGGGAACGG | | | *Xho*I |
| H9FEP-3F | GAAGACTAGTGCTATGCACTATTAACAGAGAAG | | | *Spe*I |
| H9FEP-3R | CGCCAGAGCTCCAGGTACTGTGAATCTTGCTTG | | | *Sac*I |
| J2CIR-F-Xb | GTAATGCTCTAGAGAAGCACGCACTCTGACTGGCTATGCG | | | *Xba*I |
| J2CIR-R-Nt | CATGATAGCGGCCGCGAGACCATTGTCCATTACCTGCACCAC | | | *Not*I |
| H9CIR-F-Xb | CTTTGCCTCTAGAGCTGACCTTCCGGTGGACGTGACCAACG | | | *Xba*I |
| H9CIR-R-Nt | GATACTAGGCGGCCGCCCATTGTCCATAACCTGCACCACGAG | | | *Not*I |

Underlined is incorporated nucleotides sequence for the restriction enzyme stated in the same row.

1. Kwon-Chung KJ (1976) Morphogenesis of Filobasidiella neoformans, the sexual state of Cryptococcus neoformans. Mycologia 68: 821-833.

2. Perfect JR, Lang SD, Durack DT (1980) Chronic cryptococcal meningitis: a new experimental model in rabbits. Am J Pathol 101: 177-194.
