# Supplementary material for: Iron Regulation of the Major Virulence Factors in the AIDS-Associated Pathogen Cryptococcus neoformans
Source: PLoS Biol. 2006 Nov 21;4(12):e410. doi: 10.1371/journal.pbio.0040410 (PMC1637126; doi:10.1371/journal.pbio.0040410)
Supplement: Supplementary file 2 [file pbio.0040410.st002.doc]

**Table S2. Primers used for Northern blot analysis**

| Primers | | | Sequence |
| --- | --- | --- | --- |
| Serotype D (B3501A) | FRT1 | Forward | TCTACCGCACTGGCTCTACTCTG |
|  |  | Reverse | AGTCCTCAGGAGAATCCCCATT |
|  | *FRE1* | Forward | CAAAGAGCTGTCGCTATCGCA |
|  |  | Reverse | CTTTCTTCAAAATACTCCAACGTCG |
|  | *LAC1* | Forward | GGAGCTCTCAGTATTGATAACCTGAC |
|  |  | Reverse | CGGAGAAGATGAGCGTTTTGCCGG |
|  | *SIT1* | Forward | CCGACACATGTGGGCCGATATCTTCTGG |
|  |  | Reverse | GCAACGAGCAAGGTGTAGTACAGATAATCG |
|  | *CIG1* | Forward | TTGATGGAGCCGACACCTACTACTA |
|  |  | Reverse | GATACAGACTCGTGGTCGTGCA |
|  | *ACT* | Forward | ACTTTGAGCAGGAACTCCAGACTG |
|  |  | Reverse | ACACTTTCGGTGGACGATTGAG |
| Serotype A (H99) | *FRT1* | Forward | TTAGCAACAGGAGGAAAAAGCG |
|  |  | Reverse | ATTAACCCCCCTGTTTTGGAGA |
|  | *FRE1* | Forward | CAAAGAGCTGTCGCTATCGCA |
|  |  | Reverse | CTTTCTTCAAAATACTCCAACGTCG |
|  | *LAC1* | Forward | GGAGCTCTCAGCCTTGATAACCTGAC |
|  |  | Reverse | GGAGAAGGTGAGCGTCGTGCGGGTCC |
|  | *SIT1* | Forward | CCGACACATGTGGGCCGATATCTTCTGG |
|  |  | Reverse | GCAACGAGCAAGGTGTAGTACAGATAATCG |
|  | *CIG1* | Forward | TTGATGGAGCCGACACCTACTACTA |
|  |  | Reverse | GATACAGACTCGTGGTCGTGCA |
|  | *ACT* | Forward | ACTTTGAGCAGGAACTCCAGACTG |
|  |  | Reverse | ACACTTTCGGTGGACGATTGAG |
